# Development and validation of a prediction model for early recurrence in upper tract urothelial carcinoma treated with radical nephroureterectomy

**DOI:** 10.1186/s12885-025-14180-2

**Published:** 2025-04-30

**Authors:** Yi-Ju Chou, Hao-Lun Luo, Hung-Jen Wang, Steven K. Huang, Yu-Che Hsieh, Wen-Jeng Wu, Ching-Chia Li, Han-Yu Weng, Ta-Yao Tai, Chao-Hsiang Chang, Hsi-Chin Wu, Po-Hung Lin, Jacob See-Tong Pang, Chung-Hsin Chen, Jian-Hua Hong, Jen-Shu Tseng, Marcelo Chen, I-Hsuan Alan Chen, Chia-Cheng Yu, Pi-Che Chen, Ian-Seng Cheong, Chung-You Tsai, Pai-Yu Cheng, Yuan-Hong Jiang, Yu-Khun Lee, Shian-Shiang Wang, Chuan-Shu Chen, Thomas Y. Hsueh, Wei-Chieh Chen, Chia-Chang Wu, Yung-Tai Chen, Wei-Yu Lin, Richard Chen-Yu Wu, Chi-Wen Lo, Marco Moschini, Francesco Soria, Ekaterina Laukhtina, Tamás Fazekas, Marcin Chlosta, Jeremy Yuen-Chun Teoh, Shahrokh F. Shariat, Yao-Chou Tsai

**Affiliations:** 1 Division of Urology, Department of Surgery, Taipei Tzu Chi Hospital, The Buddhist Tzu Chi Medical Foundation, New Taipei, 23142 Taiwan; 2 School of Medicine, Buddhist Tzu Chi University, Hualien, 97004 Taiwan; 3 Department of Urology, Kaohsiung Chang Gung Memorial Hospital, Kaohsiung, 83301 Taiwan; 4 School of Medicine, College of Medicine, Chang Gung University, Taoyuan, 33302 Taiwan; 5 Division of Urology, Department of Surgery, Chi Mei Medical Center, Tainan, 71004 Taiwan; 6 Department of Medical Science Industries, College of Health Sciences, Chang Jung Christian University, Tainan, 71101 Taiwan; 7 Department of Urology, Kaohsiung Medical University Hospital, Kaohsiung, 80756 Taiwan; 8 Department of Urology, School of Medicine, College of Medicine, Kaohsiung Medical University, Kaohsiung, 80708 Taiwan; 9 Graduate Institute of Clinical Medicine, College of Medicine, Kaohsiung Medical University, Kaohsiung, 80708 Taiwan; 10 Department of Urology, National Cheng Kung University Hospital, Tainan, 70101 Taiwan; 11 College of Medicine, National Cheng Kung University, Tainan, 70101 Taiwan; 12 Department of Urology, China Medical University Hospital, Taichung, 40447 Taiwan; 13 School of Medicine, China Medical University, Taichung, 40402 Taiwan; 14 Department of Urology, China Medical University Beigang Hospital, Yunlin, 65152 Taiwan; 15 Division of Urology, Department of Surgery, Chang , Gung Memorial Hospital at Linkou, Taoyuan, 33305 Taiwan; 16 Graduate Institute of Clinical Medical Science, College of Medicine, Chang Gung University, Taoyuan, 33302 Taiwan; 17 Department of Urology, National Taiwan University Hospital, Taipei, 10002 Taiwan; 18 College of Medicine, National Taiwan University, Taipei, 10002 Taiwan; 19 Institute of Biomedical Engineering, National Taiwan University, Taipei, 10617 Taiwan; 20 Department of Urology, MacKay Memorial Hospital, Taipei, 10449 Taiwan; 21 Mackay Medical College, New Taipei, 25245 Taiwan; 22 Institute of Biomedical Informatics, National Yang Ming Chiao Tung University, Taipei, 11221 Taiwan; 23 Mackay Junior College of Medicine, Nursing, and Management, Taipei, 11260 Taiwan; 24 Division of Urology, Department of Surgery, Kaohsiung Veterans General Hospital, Kaohsiung, 81362 Taiwan; 25 Department of Urology, Ditmanson Medical Foundation, Chiayi Christian Hospital, Chiayi, 60002 Taiwan; 26 Department of Surgery, Divisions of Urology, Far Eastern Memorial Hospital, New Taipei, 22060 Taiwan; 27 Department of Electrical Engineering, Yuan Ze University, Taoyuan, 32003 Taiwan; 28 Department of Urology, Hualien Tzu Chi Hospital, Buddhist Tzu Chi Medical Foundation, Hualien, 97002 Taiwan; 29 Division of Urology, Department of Surgery, Taichung Veterans General Hospital, Taichung, 40705 Taiwan; 30 Institute of Medicine, Chung Shan Medical University, Taichung, 40201 Taiwan; 31 Department of Applied Chemistry, National Chi Nan University, Nantou, 54561 Taiwan; 32 Department of Senior Citizen Service Management, National Taichung University of Science and Technology, Taichung, 40401 Taiwan; 33 Division of Urology, Department of Surgery, Taipei City Hospital Ren-Ai Branch, Taipei, 10629 Taiwan; 34 Department of Urology, School of Medicine, National Yang Ming Chiao Tung University, Taipei, 11221 Taiwan; 35 Department of Urology, Taipei Medical University Hospital, Taipei Medical University, Taipei, 11031 Taiwan; 36 Department of Urology, Shuang Ho Hospital, Taipei Medical University, New Taipei, 23561 Taiwan; 37 Department of Urology, School of Medicine, College of Medicine, Taipei Medical University, Taipei, 11031 Taiwan; 38 TMU Research Center of Urology and Kidney (TMU-RCUK), Taipei Medical University, Taipei, 11031 Taiwan; 39 Department of Urology, Postal Hospital, Taipei, 10078 Taiwan; 40 40Department of Urology, Taiwan , Adventist Hospital, Taipei, 10556 Taiwan; 41 Division of Urology, Department of Surgery, Chang Gung Memorial Hospital, Chia-Yi, 61363 Taiwan; 42 Chang Gung University of Science and Technology, Chia-Yi, 61363 Taiwan; 43 Department of Urology, E-Da Hospital, Kaohsiung, 82445 Taiwan; 44 Department of Information Engineering, I-Shou University, Kaohsiung, 84001 Taiwan; 45 Department of Urology, IRCCS San Raffaele Hospital and Vita-Salute San Raffaele University, Milan, Italy; 46 Division of Urology, Department of Surgical Sciences, San Giovanni Battista Hospital, University of Studies of Torino, Turin, Italy; 47 Department of Urology, Comprehensive Cancer Center, Medical University of Vienna, Vienna, Austria; 48 Department of Urology, Semmelweis University, Budapest, Hungary; 49 Clinic of Urology and Urological Oncology, Jagiellonian University, Krakow, Poland; 50 Department of Surgery, S.H. Ho Urology Centre, The Chinese University of Hong Kong, Hong Kong, China; 51 Department of Urology, The University of Texas Southwestern Medical Center at Dallas, Dallas, TX USA; 52 Hourani Center for Applied Scientific Research, Al-Ahliyya Amman University, Amman, Jordan; 53 Karl Landsteiner Institute of Urology and Andrology, Vienna, Austria; 54 Department of Urology, Weill Cornell Medical College, New York, NY USA

**Keywords:** Upper tract urothelial carcinoma, Early recurrence, Risk factor, Prediction model

## Abstract

**Background:**

Most cases of upper tract urothelial carcinoma (UTUC) exhibit recurrence within the first year following surgery. The time from surgery to recurrence significantly impacts cancer-specific survival. In this study, we analyzed patients with localized UTUC (pTis–3N0/xcM0) who experienced postoperative recurrence to identify an appropriate early recurrence time point and the associated risk factors.

**Methods:**

From July 1988 to October 2022, we retrospectively analyzed 3435 localized UTUC patients after undergoing radical nephroureterectomy using Taiwan's UTUC Collaboration Group Database. Early recurrence time point was defined according to oncologic outcome. Variables including clinical and pathological characteristics were assessed in relation to early recurrence. A prediction model was constructed by factors associated with early recurrence and externally validated.

**Results:**

Early recurrence time point in localized UTUC was determined at 9 months post-surgery, with patients experiencing early recurrence exhibiting worse overall and cancer specific survival. Diabetes mellitus, multifocality, lympho-vascular invasion, tumor necrosis and pathologic T stage were independent factors associated with early recurrence. The predictive model for early recurrence achieved an area under the curve (AUC) of 0.84 (95%CI: 0.82–0.86). External validation demonstrated that the model exhibited good discrimination (AUC: 0.76, 95%CI: 0.73–0.79), calibration (Brier score: 0.08) and clinical utility in a distinct cohort.

**Conclusions:**

This study identified the optimal time point for early recurrence and its associated risk factors. A prediction model for early recurrence was developed based on these factors and validated externally, demonstrating good generalizability. This clinical tool can facilitate early identification of high-risk patients, enabling targeted surveillance and timely intervention. Future studies should explore effective treatment strategies for preventing early recurrence.

**Supplementary Information:**

The online version contains supplementary material available at 10.1186/s12885-025-14180-2.

## Introduction

Compared to Western countries, upper tract urothelial carcinoma (UTUC) is more prevalent in Taiwan. While UTUC accounts for only 5–10% of all urothelial carcinomas in Western nations, it comprises 30% of cases in Taiwan [1, 2]. The incidence rate of UTUC in Taiwan stands at approximately 3.1–3.4 per 100,000 person-years, whereas in Western countries, it is about 1.1–1.4 per 100,000 person-years [3, 4]. These figures highlight a markedly higher incidence of UTUC in Taiwan. Exposure to risk factors such as Chinese herbal medicines containing aristolochic acid and consumption of arsenic-contaminated groundwater has contributed to this elevated incidence of UTUC [5, 6]. This, in turn, provides us with a substantial number of patients to gain a deeper understanding of this cancer.

Prior studies have demonstrated that the median time to recurrence of UTUC post-surgery ranged from 10.4 to 15 months [7, 8]. These recurrent patients have a low chance of surviving beyond three years, with a three-year cancer-specific survival (CSS) rate of only 9.7%. To better understand the characteristics of recurrent patients and provide appropriate treatment to improve their prognosis, many studies have extensively investigated recurrence [7, 9, 10]. Age, adverse pathological features, and tumor architecture are common factors associated with recurrence. Models built using these risk factors can accurately predict recurrence, enabling these patients to receive suitable adjuvant therapy [11].

While substantial knowledge exists about recurrent patients, few studies have explored whether the duration from surgery to recurrence influences oncologic outcomes. One study revealed that shorter intervals between surgery and recurrence are linked to poorer survival outcomes post-recurrence [12]. Patients experiencing recurrence within the initial 12 months exhibit worse CSS compared to those with later recurrences. This implies that early recurrence patients may represent a distinct subgroup with inferior prognoses. Consequently, this study aims to determine the optimal early recurrence definition, identify key risk factors, and develop a predictive model to guide clinical decision-making. This model can help identify the distinct subgroup of patients prone to early recurrence, allowing for tailored surveillance strategies and adjuvant treatments.

## Materials and methods

### Study population

The study population was derived from the Taiwan Upper Tract Urothelial Carcinoma Collaboration Group Database. This database collected clinical and pathological characteristics of patients with UTUC from 21 hospitals across Taiwan. All patients were de-identified, thus informed consent was not required. As of October 2022, a total of 5,571 patients were available for analysis. This retrospective data analysis was performed in accordance with the Declaration of Helsinki and was approved by the Institutional Review Board of Taipei Tzu Chi Hospital (No. 06-X34-105). The requirement for informed consents was waived by the Institutional Review Board of Taipei Tzu Chi Hospital due to the retrospective nature of this study.

From July 1988 to October 2022, patients with localized UTUC, no history of neoadjuvant chemotherapy, and no concurrent bladder cancer were selected for analysis. Localized UTUC was defined as those with staging of pTis–3N0/xcM0. After excluding patients who did not meet the inclusion criteria, a total of 2,839 patients without recurrence and 689 patients with recurrence were identified. Among the patients who experienced recurrence, 93 patients who recurred within one month after surgery were further excluded, leaving a total of 596 patients eligible for analysis of early recurrence (Supplement Fig. 1). All included patients underwent radical nephroureterectomy and bladder cuff excision. Lymph node dissection was performed only for patients suspected of having lymph node metastasis, and the extent of lymph node dissection was determined by the operating surgeon. The administration and regimen of adjuvant chemotherapy were determined by the treating physician based on the patient’s clinical or pathological stage and general health status.

### Analysis variables and definitions

A total of 17 variables were selected to investigate their association with early recurrence. The performance status of patients was assessed and scored according to the Eastern Cooperative Oncology Group (ECOG) criteria. Tumor involvement of the affected side and preoperative hydronephrosis were evaluated using cross sectional imaging. All resected specimens were analyzed by pathologists at each participating hospital. Tumor location was classified as involving the ureter or renal pelvis. Multifocality was defined as the presence of tumors in more than one location or the concurrent presence of carcinoma in situ. Tumor grade and pathological stage were determined based on the 2004 World Health Organization grading system and the 2017 TNM staging system of the American Joint Committee on Cancer, respectively.

### Analysis of oncologic outcome and definitions

Recurrence was confirmed by cross sectional imaging and/or pathological examination, which included local recurrence within the tumor bed, lymph node, or distant metastasis. Urothelial cancer occurring in the bladder or the contralateral upper tract after surgery was not considered a recurrence. Recurrence-free survival (RFS) was defined as the time from surgery to first recurrence. Overall survival (OS) and CSS were defined as the time from surgery to death from any cause and death specifically attributed to cancer, respectively. The cause of death was primarily determined based on death certificates, and in cases of uncertainty, medical records were reviewed to ascertain the cause of death.

### Follow-up protocols

In general, patients were evaluated every 3–6 months through medical history, physical examination, urine cytology, renal ultrasound, and cystoscopy. Abdominal computed tomography or magnetic resonance imaging was conducted every 6–12 months to evaluate for recurrence. If clinically suspected, additional chest computed tomography or bone scan was arranged to assess for distant metastasis.

### External validation cohort

Patients from a UTUC database comprising 16 centers distributed across Europe, North America, and Hong Kong were used for external validation [13]. The validation cohort was selected according to the inclusion and exclusion criteria of the present study. A total of 538 patients with recurrence and 1,708 patients without recurrence were included for external validation.

### Statistical analysis

Categorical variables were expressed as percentages and compared using the Chi-square test. The optimal time point for early recurrence was determined based on the minimum p-value approach [14]. The log-rank test was utilized to compare the CSS of recurrent patient groups divided according to different recurrence time points. The time point associated with the lowest p-value was defined as the"early recurrence"time point. Univariate and multivariate Cox regression analyses were used to evaluate clinical and pathological variables’ association with OS and CSS. Univariable logistic regression was performed to assess the association between clinical and pathological variables and early recurrence. Variables that showed a significant association with early recurrence in the univariable analysis were included in the multivariable analysis. Only independent risk factors that remained significant in the multivariable analysis were incorporated into the early recurrence prediction model, and a receiver operating characteristic (ROC) curve was constructed for this model. Both patients with and without recurrence were included in the development of the prediction model. The Kaplan–Meier curve was employed to depict the relationship between OS, CSS, and RFS among different groups, and comparisons were made using the Cox proportional hazards model. After the establishment of the early recurrence prediction model, its predictive accuracy was evaluated in the multicenter validation cohort in terms of discrimination, calibration, and clinical utility [15]. The discriminative ability of the model was quantified using the area under the ROC curve. Calibration plot and Brier score were employed to represent the relationship between the model-predicted risk and the observed risk. Decision curve analysis was used to assess the clinical utility of the model [16]. All statistical analyses were two-tailed, and statistical significance was considered at p < 0.05. The R software version 4.3.3 (R Foundation for Statistical Computing, Vienna, Austria) was used for all statistical analyses.

## Results

### Definition of the early recurrence time point in localized UTUC

The lowest p-value for the difference in CSS between the early and late recurrence groups was found at 9 months. Therefore, the early recurrence time point in localized UTUC was defined as within 9 months after surgery (Supplement Fig. 2). Based on this recurrence time point, the patient cohort was divided into early and late recurrence groups, consisting of 231 and 365 individuals, respectively (Supplement Fig. 1).

### Early versus late recurrence

**Fig. 1:**
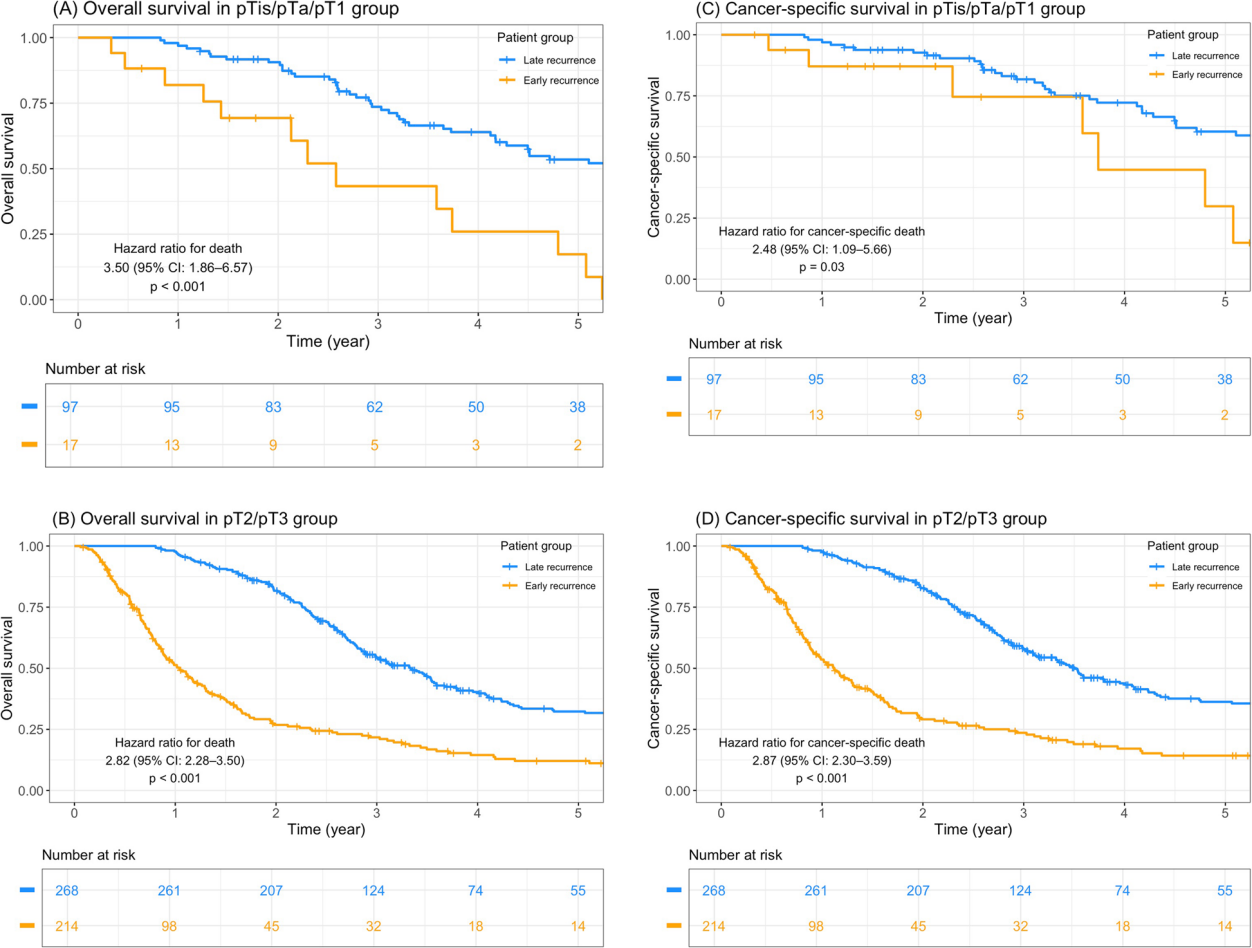
Oncologic outcome of early and late recurrence group. **A** Overall survival in pTis/pTa/pT1 group. **B** Overall survival in pT2/pT3 group. **C** Cancer-specific survival in pTis/pTa/pT1 group. **D** Cancer-specific survival in pT2/pT3 group

Table 1 presented the clinical and pathological characteristics of the two patient groups. Regarding clinical features, significant differences were observed in terms of presence of diabetes mellitus (DM) and intravesical recurrence. Significant pathological differences were noted, including tumor location, histological grade, lympho-vascular invasion (LVI), tumor necrosis (TN), and pathologic T stage. Patients with early recurrence exhibited inferior OS and CSS compared to those with late recurrence, regardless of low or high pathologic T stage (Fig. 1). In the multivariate Cox regression model, early recurrence was a significant factor influencing OS and CSS (Supplement Table 1).

**Table 1:** Clinical and pathologic characteristics of early and late recurrence group

Variables	Early recurrence (*N* = 231)		Late recurrence (*N* = 365)		*p*-value
	N	%	N	%	
Age					
< 70	103	44.6	192	52.6	0.056
≥ 70	128	55.4	173	47.4	
Sex					
Male	109	47.2	164	44.9	0.590
Female	122	52.8	201	55.1	
ECOG					
0	128	55.4	236	64.7	0.081
1	81	35.1	110	30.1	
2	20	8.7	17	4.7	
3	1	0.4	2	0.5	
4	1	0.4	0	0.0	
DM					
No	157	68.0	281	77.0	0.015
Yes	74	32.0	84	23.0	
ESRD/Renal insufficiency					
No	90	39.0	158	43.3	0.296
Yes	141	61.0	207	56.7	
Smoking					
No	183	79.2	297	81.4	0.518
Yes	48	20.8	68	18.6	
Tumor side					
Unilateral	229	99.1	363	99.5	0.643
Bilateral	2	0.9	2	0.5	
Preoperative hydronephrosis					
No	82	35.5	137	37.5	0.615
Yes	149	64.5	228	62.5	
Tumor location					
Ureter	74	32.0	152	41.6	0.018
Renal pelvis	157	68.0	213	58.4	
Grade					
Low grade	7	3.0	27	7.4	0.025
High grade	224	97.0	338	92.6	
Multifocality					
No	64	27.7	88	24.1	0.326
Yes	167	72.3	277	75.9	
Lympho-vascular invasion					
No	124	53.7	286	78.4	< 0.001
Yes	107	46.3	79	21.6	
Tumor necrosis					
No	167	72.3	302	82.7	0.002
Yes	64	27.7	63	17.3	
Pathologic T stage					
pTis/pTa/pT1	17	7.4	97	26.6	< 0.001
pT2	37	16.0	76	20.8	
pT3	177	76.6	192	52.6	
Lymph node dissection					
No	176	76.2	301	82.5	0.061
Yes	55	23.8	64	17.5	
Adjuvant chemotherapy					
No	195	84.4	293	80.3	0.201
Yes	36	15.6	72	19.7	
Regimen of adjuvant chemotherapy					
Cisplatin-based	11	30.6	20	27.8	0.867
Carboplatin-based	10	27.8	24	33.3	
MVAC	5	13.9	12	16.7	
Others	10	27.8	16	22.2	
Intravesical recurrence					
No	120	51.9	179	49.1	< 0.001
Yes	45	19.5	146	40.0	
Unknown	66	28.6	40	10.9	
Upper tract extra-urothelial recurrence					
No	142	61.5	252	69.1	0.062
Yes	44	19.0	45	12.3	
Unknown	45	19.5	68	18.6	
Lower tract extra-urothelial recurrence					
No	156	67.5	263	72.1	0.180
Yes	32	13.9	33	9.0	
Unknown	43	18.6	69	18.9	
Lymph node recurrence					
No	100	43.3	175	47.9	0.537
Yes	89	38.5	130	35.6	
Unknown	42	18.2	60	16.4	
Distant metastasis					
No	55	23.8	105	28.8	0.274
Yes	161	69.7	231	63.3	
Unknown	15	6.5	29	7.9

### Identifying risk factors for early recurrence

A total of 17 factors were selected to investigate their predictive value for early recurrence (Table 2). Univariate analysis revealed that 11 factors were significantly associated with early recurrence, including age, performance status, presence of DM, smoking, preoperative hydronephrosis, tumor location, tumor grade, multifocality, LVI, TN, and pathologic T stage. Subsequently, multivariate analysis of these factors identified 5 factors that remained significantly associated with early recurrence, namely presence of DM, multifocality, LVI, TN and pathologic T stage.

**Table 2:** Exploring risk factors for early recurrence using univariate and multivariate logistic regression

Variables	Univariate			Multivariate		
	OR	95% CI	*p*-value	OR	95% CI	*p*-value
Age	1.38	1.14–1.68	0.001	1.21	0.98–1.48	0.072
Sex						
Male	Reference					
Female	0.79	0.60–1.03	0.084			
ECOG						
0–1	Reference			Reference		
2–4	2.02	1.26–3.22	0.003	1.64	0.96–2.80	0.071
DM						
No	Reference			Reference		
Yes	1.67	1.25–2.23	< 0.001	1.40	1.01–1.93	0.041
ESRD/Renal insufficiency						
No	Reference					
Yes	0.98	0.75–1.29	0.883			
Smoking						
No	Reference			Reference		
Yes	1.46	1.05–2.03	0.025	1.41	0.98–2.03	0.067
Tumor side						
Unilateral	Reference					
Bilateral	1.11	0.26–4.72	0.887			
Preoperative hydronephrosis						
No	Reference			Reference		
Yes	1.80	1.37–2.38	< 0.001	1.34	0.98–1.82	0.065
Tumor location						
Ureter	Reference			Reference		
Renal pelvis	1.36	1.02–1.80	0.036	1.20	0.87–1.66	0.261
Tumor grade						
Low grade	Reference			Reference		
High grade	5.57	2.61–11.89	< 0.001	1.68	0.76–3.73	0.199
Multifocality						
No	Reference			Reference		
Yes	3.45	2.57–4.65	< 0.001	2.91	2.12–3.98	< 0.001
Lympho-vascular invasion						
No	Reference			Reference		
Yes	5.60	4.24–7.39	< 0.001	2.52	1.86–3.41	< 0.001
Tumor necrosis						
No	Reference			Reference		
Yes	2.81	2.06–3.82	< 0.001	1.78	1.27–2.50	< 0.001
Pathologic T stage						
pTis/pTa/pT1	Reference			Reference		
pT2	5.34	2.99–9.55	< 0.001	4.12	2.26–7.49	< 0.001
pT3	17.58	10.62–20.10	< 0.001	9.90	5.82–16.85	< 0.001
Lymph node dissection						
No	Reference					
Yes	1.27	0.93–1.74	0.138			
Adjuvant chemotherapy						
No	Reference					
Yes	1.31	0.90–1.89	0.158			

### Predictive model for early recurrence

**Fig. 2:**
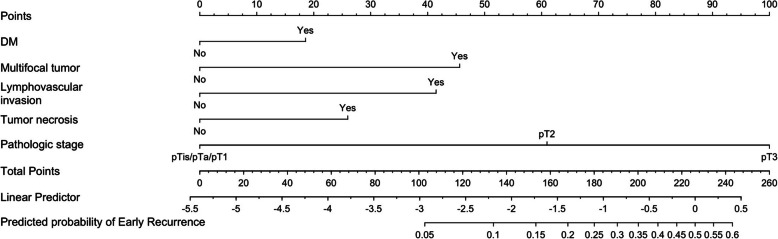
The nomogram for predicting early recurrence

**Fig. 3:**
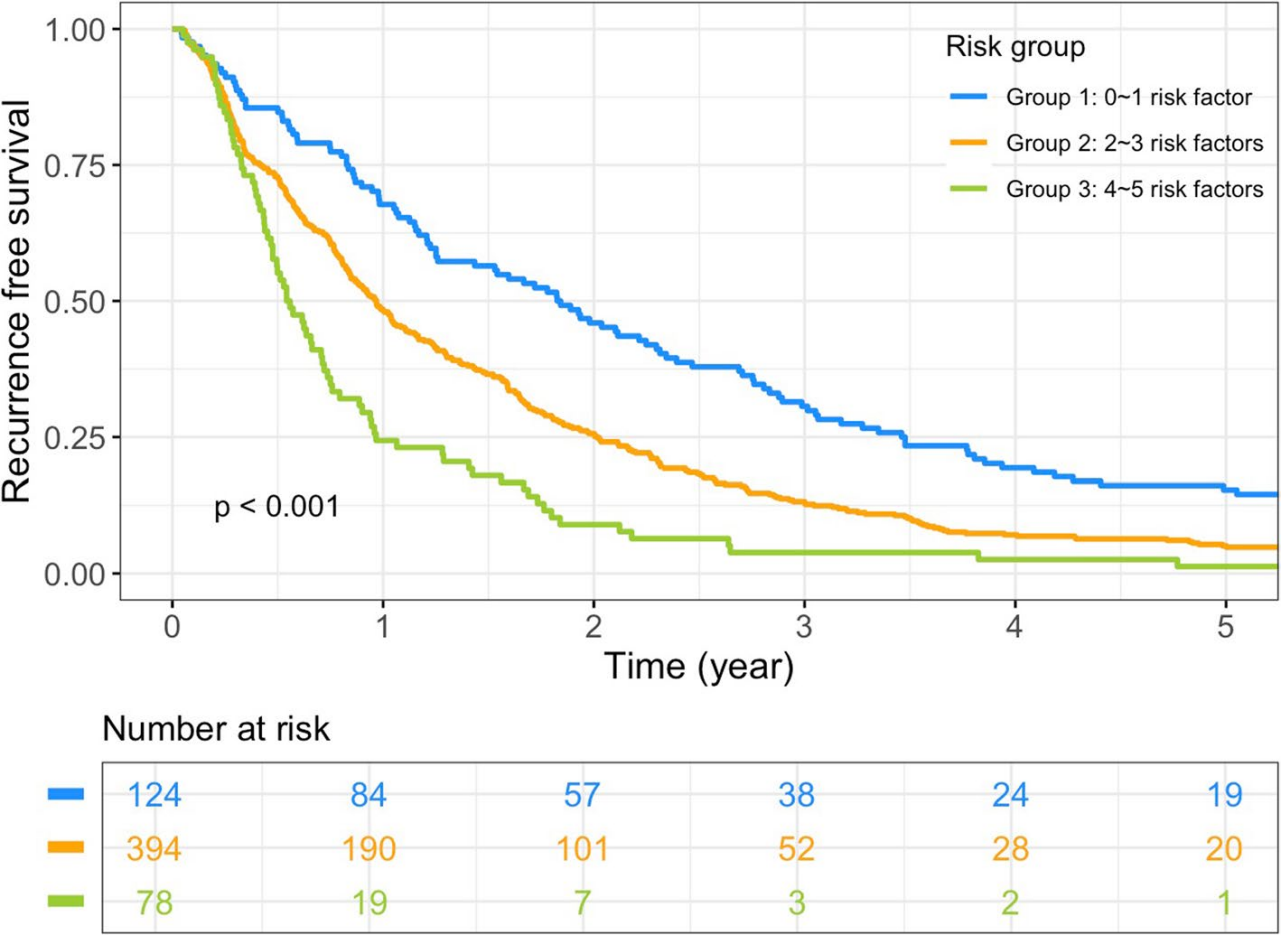
Recurrence free survival between groups stratified according to numbers of risk factors for early recurrence

A predictive model for early recurrence in localized UTUC was developed based on the 5 independent factors identified in the multivariate analysis. The final model is expressed as follows: $\text{log}(\frac{\text{P}}{1-\text{P}})$= − 5.3951 + 0.4428 × (DM = Yes) + 1.0885 × (Multifocality = Yes) + 0.9899 × (LVI = Yes) + 0.6198 × (TN = Yes) + 1.4543 × (pT2 = Yes) + 2.3863 × (pT3 = Yes), where P represents the predicted probability of early recurrence. The ROC curve in Supplement Fig. 3 demonstrated an area under the curve (AUC) of 0.84 (95% CI: 0.82–0.86, p < 0.001) for this predictive model in forecasting early recurrence. Figure 2 presents the nomogram constructed based on the predictive model. Patients were classified into three groups based on the number of risk factors present: Group 1 with 0–1 risk factor, Group 2 with 2–3 risk factors, and Group 3 with 4–5 risk factors. Figure 3 illustrated significant differences in RFS among these three patient groups. In addition, an increased number of risk factors was associated with poorer OS and CSS (Supplement Fig. 4).

### External validation of the prediction model

**Fig. 4:**
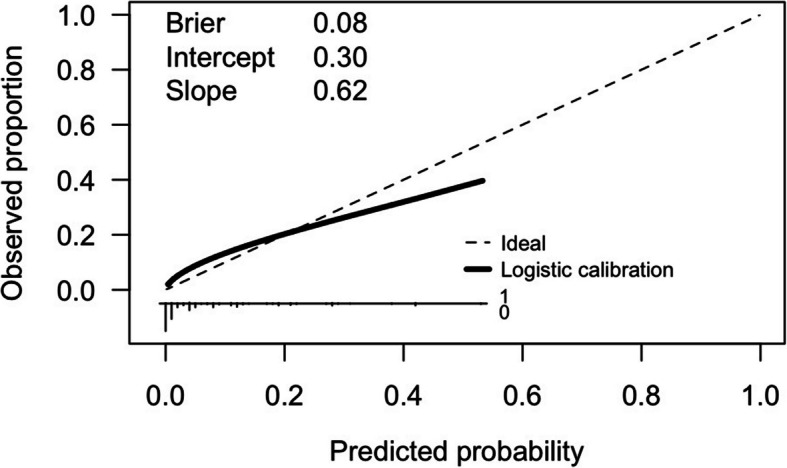
Calibration plot of the early recurrence prediction model on the validation cohort

**Fig. 5:**
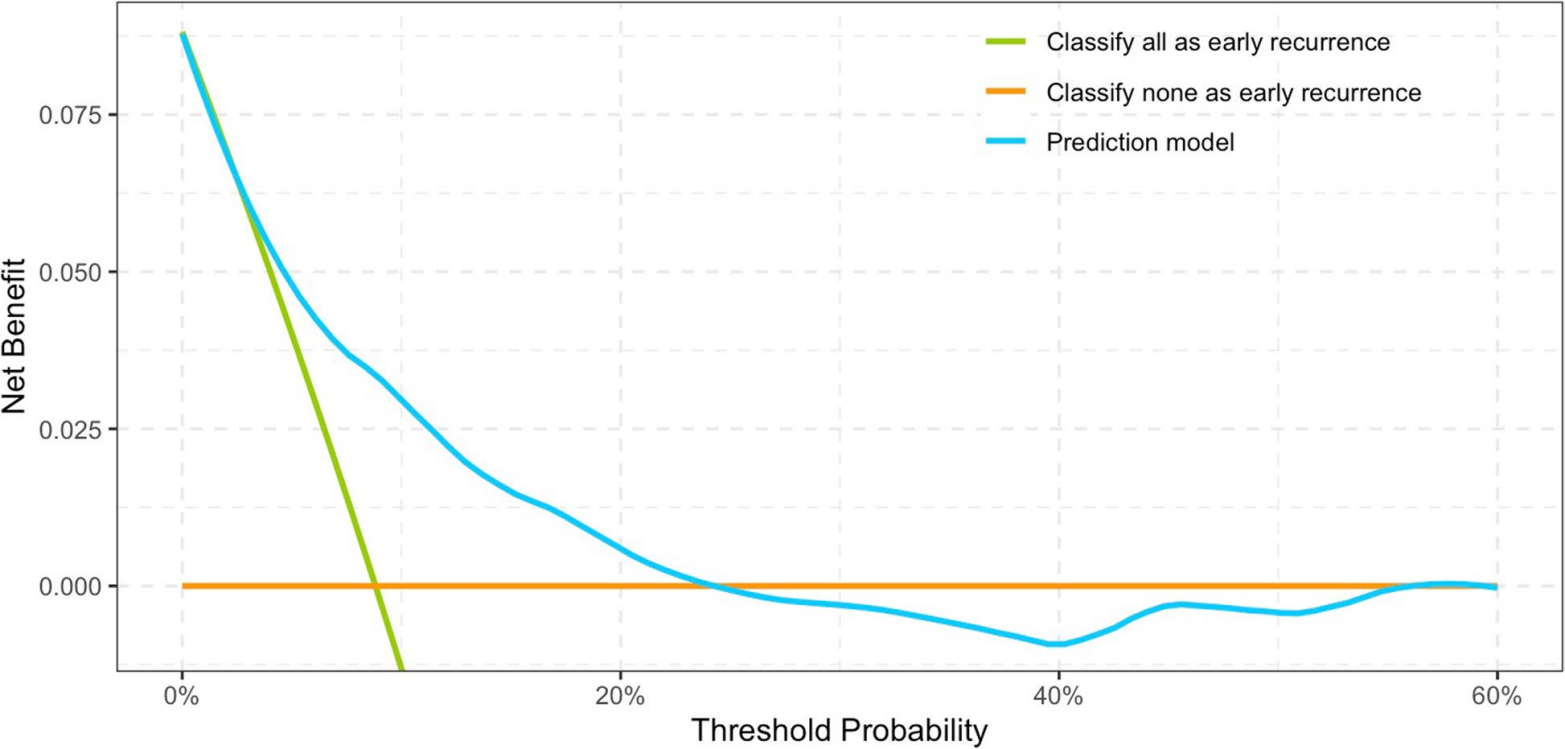
Decision curve of the early recurrence prediction model on the validation cohort

Compared to the development cohort, the proportion of early recurrence was higher in the validation cohort. The two groups were comparable in terms of characteristics such as age, smoking history, tumor location, tumor grade, and pathologic T stage. However, significant differences between the two cohorts were observed in sex, ECOG status, DM, tumor grade, multifocality, LVI, TN, lymph node dissection, and adjuvant chemotherapy (Supplement Table 2). The prediction model applied to the validation cohort has an AUC of 0.76 (95% CI: 0.73–0.79) (Supplement Fig. 3). The calibration plot shows good calibration, with a Brier score of 0.08 (Fig. 4). Decision curve analysis indicates a positive net benefit for this model at a threshold probability ranging from 3 to 24% (Fig. 5).

## Discussion

Numerous studies have identified prognostic factors for oncologic outcomes in UTUC, enabling personalized surveillance and treatment strategies [7, 17]. However, research specifically addressing early recurrence in UTUC remains limited, and little is known about this patient subgroup. Our study defined early recurrence as recurrence within 9 months post-surgery and found significantly worse OS and CSS outcomes in these patients after multivariate analysis. Independent predictors of early recurrence included presence of DM, multifocality, LVI, TN, and advanced pathologic T stage. An increased number of risk factors was associated not only with earlier recurrence but also with worse OS and CSS. We constructed and externally validated a predictive model with good discrimination, calibration, and clinical utility. A corresponding nomogram was developed to help clinicians stratify patients for closer monitoring and timely interventions to improve patient outcomes.

Given the elevated occurrence of bladder UC, there has been research directed towards comprehending the early recurrence patterns within bladder UC [18]. The study revealed a clear association between the duration from surgery to recurrence and cancer-specific mortality and risk factors related to early recurrence were analyzed. Despite a similar association of time to recurrence with cancer-specific mortality in the field of UTUC, an investigation into early recurrence has been lacking [12]. This could result in the application of uniform follow-up and treatment strategies to populations with differing prognoses. Hence, there is an urgent need for research focused on early recurrence in UTUC.

The early recurrence study in bladder UC employed a subjective definition of two years as the early recurrence time cutoff, without anchoring it to oncologic outcomes. Multivariate analysis in the study revealed a correlation between higher pathologic T stage and early recurrence [18]. However, the study explored only eight risk factors and did not incorporate crucial pathological findings into their analyses. In the present investigation, we have embraced a methodology akin to early recurrence studies in diverse cancers, employing the minimum p-value approach to pinpoint a 9-month threshold for early recurrence within UTUC [14]. Our study encompasses a wider array of clinical and pathological attributes to examine their correlation with early recurrence. This approach not only augments our understanding of this patient cohort but also furnishes the groundwork for developing predictive models aimed at enhancing prognostic accuracy.

Several nomograms have been developed to predict postoperative recurrence in UTUC, although each incorporates different predictive variables [9, 11, 19]. Similar to these existing nomograms, our study also identified pathologic T stage and LVI as significant risk factors. However, unlike prior studies, age and performance status were not significantly associated with early recurrence in our analysis. Instead, the most relevant risk factors for early recurrence were predominantly adverse pathological features reflecting tumor aggressiveness, suggesting that intrinsic tumor biology may play a greater role in determining early recurrence. Furthermore, previous nomograms, despite external validation, have generally used validation cohorts randomly selected from the original study populations rather than genuinely distinct patient groups. This may limit their accuracy when applied to different populations. The nomogram developed in our study underwent external validation using a completely different, ethnically diverse cohort, demonstrating good predictive performance for early recurrence. Consequently, this nomogram provides enhanced clinical applicability and greater generalizability.

In this study, DM was identified as a significant risk factor for early recurrence. Previous study has primarily associated DM with bladder recurrence; however, after considering glycemic control, poor glucose management has been linked to worse oncologic outcomes [20, 21]. Patients with well-controlled blood glucose levels have comparable oncologic outcomes to those without diabetes [21]. The negative prognostic effect of DM may be attributed to hyperglycemia, hyperinsulinemia, and chronic inflammation, as hormonal imbalance and inflammation can promote cell proliferation and tumor progression [22]. Therefore, given that DM is the only modifiable risk factor for early recurrence identified in this study, ensuring adequate preoperative glycemic control in UTUC patients is crucial.

Apart from DM, all other risk factors associated with early recurrence were adverse pathological features indicative of aggressive tumor behavior and have previously been linked to poor oncologic outcomes [23]. Multifocality and LVI have been associated with worse RFS, whereas TN, although not independently associated with worse RFS, often coexists with other adverse pathological characteristics related to decreased survival [24–26]. Our study demonstrates that these risk factors not only increase recurrence rates but also substantially elevate the risk of early recurrence postoperatively. Thus, immediate postoperative adjuvant therapy might be critical in improving RFS for patients exhibiting these risk factors.

The POUT trial showed that adjuvant chemotherapy significantly improved disease-free survival and OS compared to surveillance in patients with muscle-invasive UTUC [27]. However, our analysis did not reveal a protective effect of adjuvant chemotherapy against early recurrence or mortality. One possible explanation is that not all patients in our cohort received guideline-recommended standard chemotherapy regimens. Another potential reason is that patients who experienced early recurrence may have intrinsic resistance to chemotherapy. In recent studies on metastatic urothelial carcinoma, treatment options with better efficacy than standard chemotherapy have been identified [28, 29]. It is possible that combining chemotherapy with immunotherapy or using novel antibody–drug conjugates may be more effective in preventing early recurrence. Therefore, future studies are needed to explore appropriate adjuvant therapies for the prevention of early recurrence.

This study still had some limitations. First, the retrospective study design carries the potential for selection bias. The heterogeneity in surgical approaches due to incorporation of multi-center data has led to variations in the implementation of lymph node dissection by different surgeons. Patients lacking lymph node for pathologic assessment might underestimate disease severity. However, we have excluded patients who experienced recurrence within one month after surgery to prevent cases with pre-existing metastasis from being mistakenly classified as early recurrence. Second, adjuvant chemotherapy regimens were also not standardized due to the retrospective design, making it difficult to evaluate the precise role of adjuvant therapy in preventing early recurrence. Third, all pathology specimens were analyzed by respective hospital pathologists, and the absence of central pathologic review poses a validation concern. Fourth, information regarding individual surgeons'experience was unavailable, precluding analysis of surgeon experience on early recurrence outcomes. Despite these limitations, the main strength of this study is the creation of a prediction tool for early recurrence in patients with UTUC. This risk assessment model may aid clinical decision-making regarding recommendations for adjuvant therapy or intensified postoperative monitoring. Future research should aim to refine this predictive model by incorporating additional variables associated with early recurrence and validating its performance in prospective, randomized cohorts. Moreover, exploring the use of advanced surveillance methods, such as circulating tumor DNA, as well as identifying effective adjuvant treatments, will be essential for improving early detection and preventing recurrence in these high-risk patients [30].

## Conclusions

We identified that recurrence within 9 months post-surgery is the optimal time point for early recurrence in UTUC patients. Through the incorporation of risk factors such as DM, multifocality, LVI, TN, and pathologic T stage, we successfully developed and externally validated a model capable of predicting early recurrence. Through this model, we can successfully identify the subgroup of patients with early recurrence who have poorer prognoses, allowing for closer surveillance and timely adjuvant therapy. However, effective preventive measures for early recurrence remain unknown; thus, further studies are necessary to explore effective treatment strategies.

## Supplementary Information

Supplementary Material 1.

## Data Availability

The data sets generated during and/or analyzed during the current study are available from the corresponding author on reasonable request.
