# Supplementary material for: Development and validation of a prediction model for early recurrence in upper tract urothelial carcinoma treated with radical nephroureterectomy
Source: BMC Cancer. 2025 Apr 30;25:808. doi: 10.1186/s12885-025-14180-2 (PMC12042504; doi:10.1186/s12885-025-14180-2)
Supplement: Supplementary file 1 — Supplementary Material 1. [file 12885_2025_14180_MOESM1_ESM.docx]

**Supplementary materials**

**Supplement figure 1 Study flow chart**

**
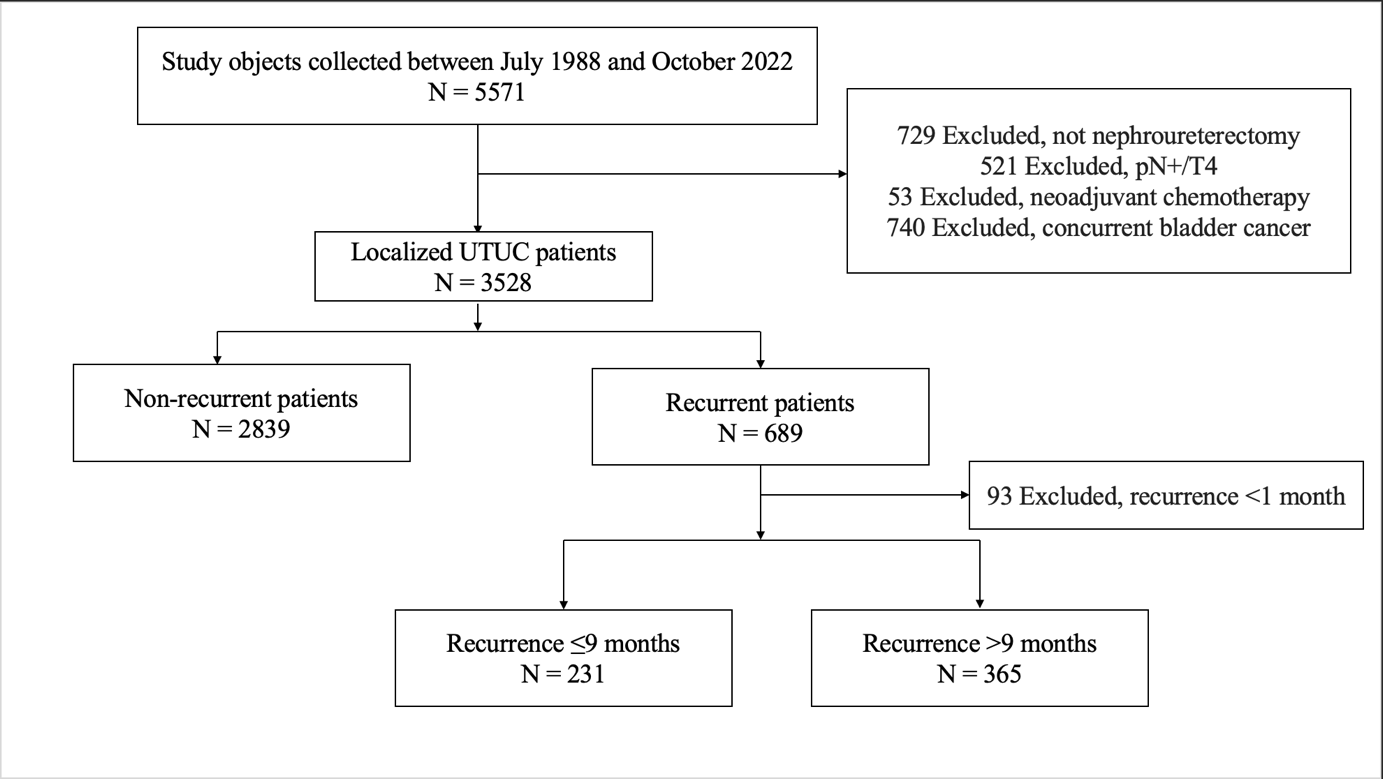
**

**Supplement figure 2 Different cutoff threshold of early recurrence and their corresponding p-value**

**
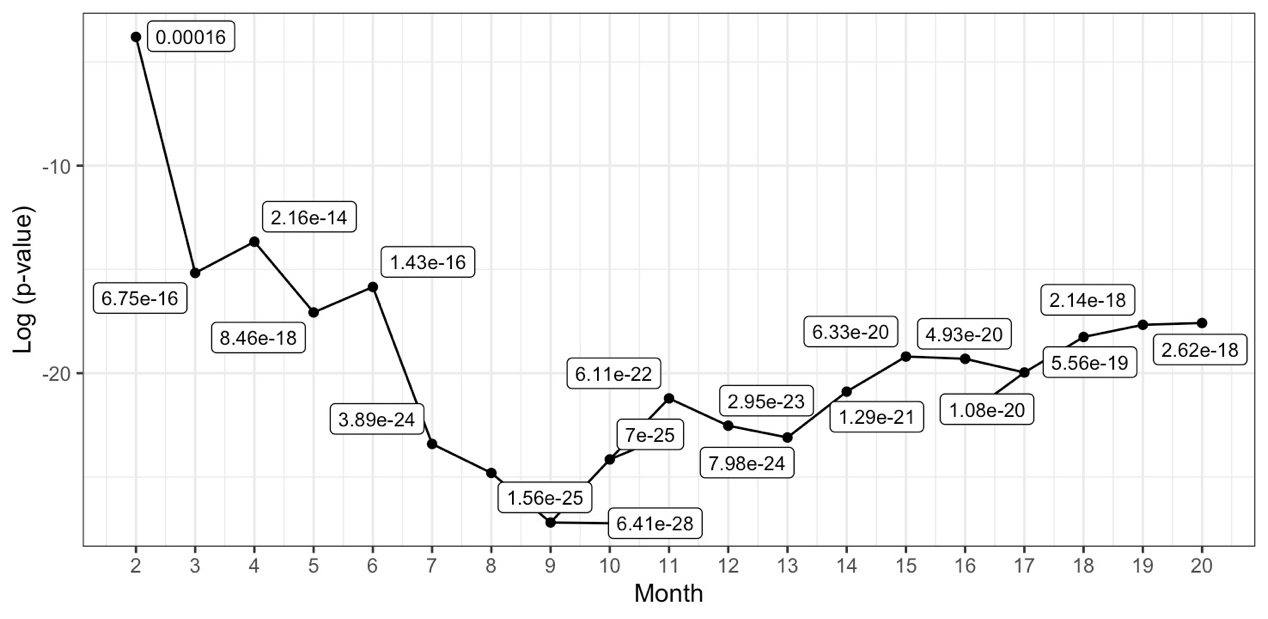
**

**Supplement figure 3 ROC curve of the prediction model for early recurrence**

**(A) Development cohort**

**
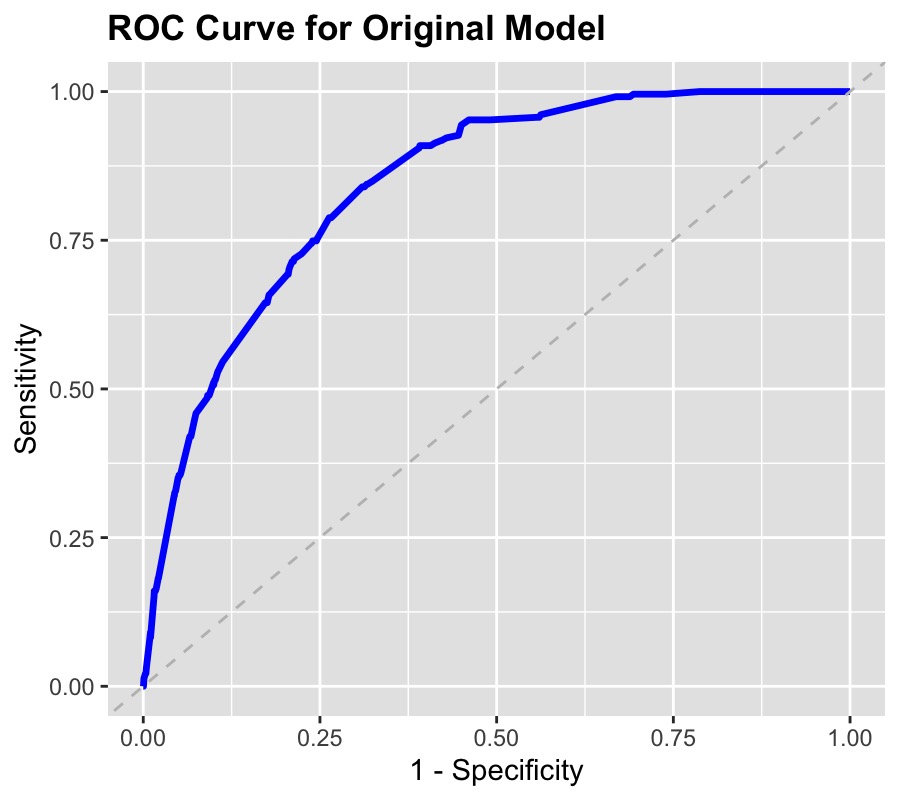
**

**(B) Validation cohort**

**
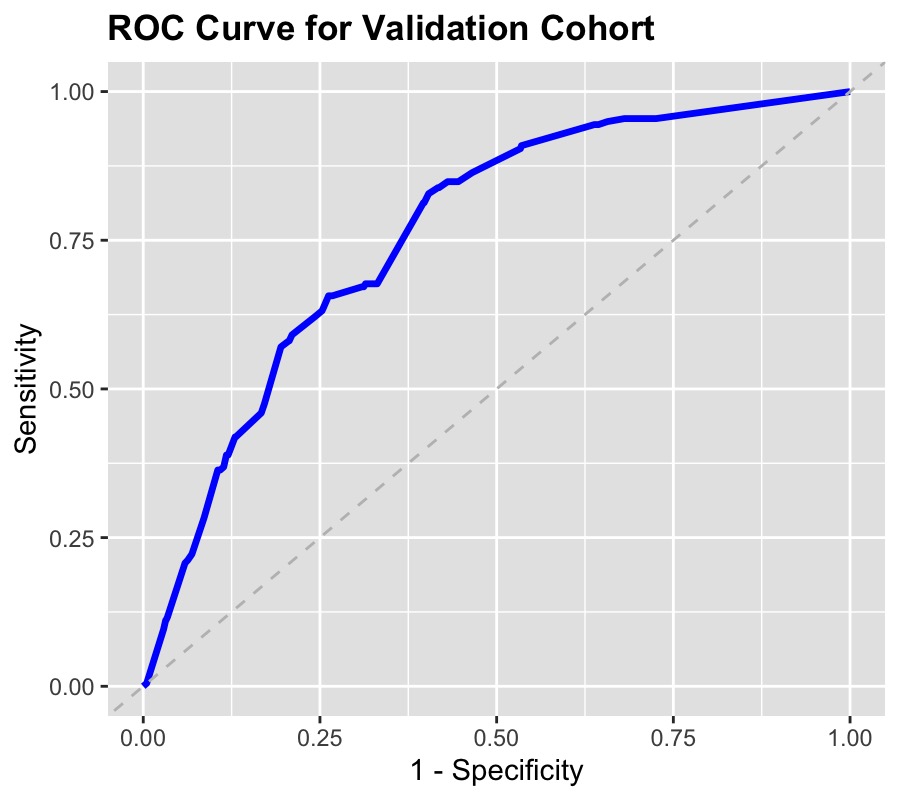
**

**Supplement Figure 4 Survival between groups stratified according to numbers of risk factors for early recurrence**

1. **Overall survival**

**
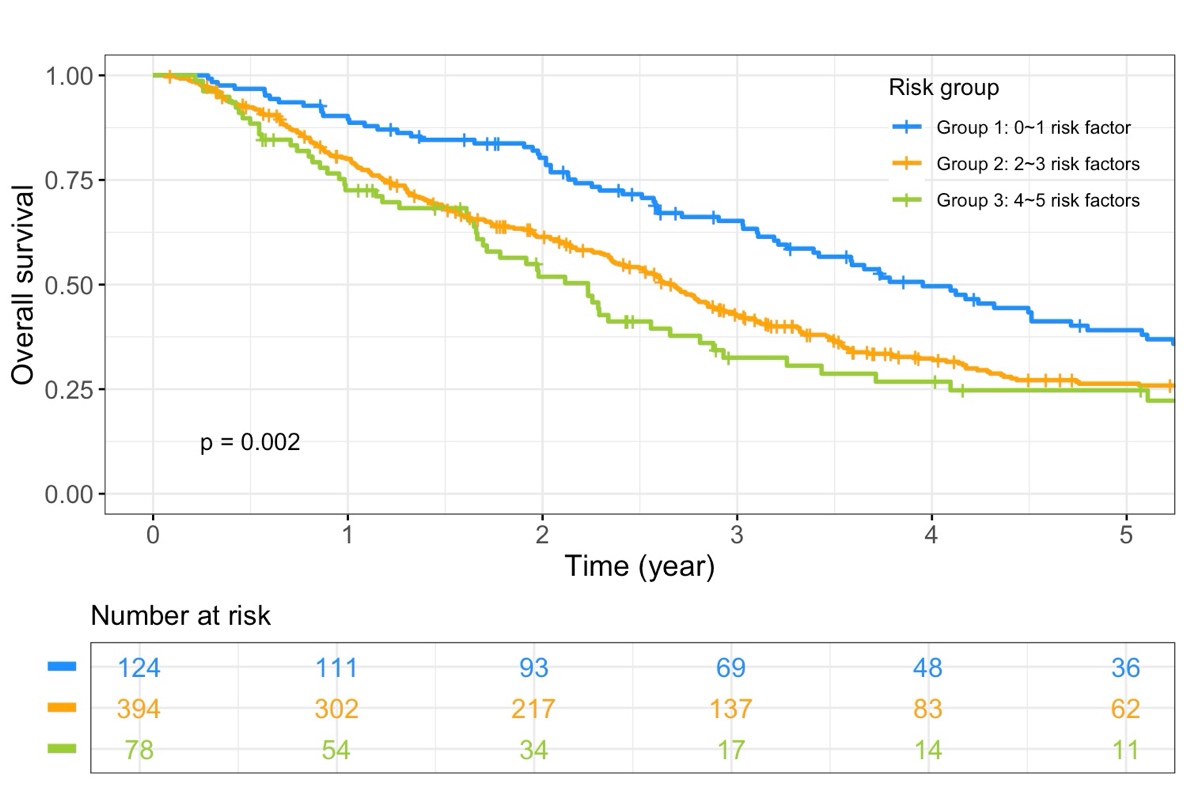
**

1. **Cancer-specific survival**

**
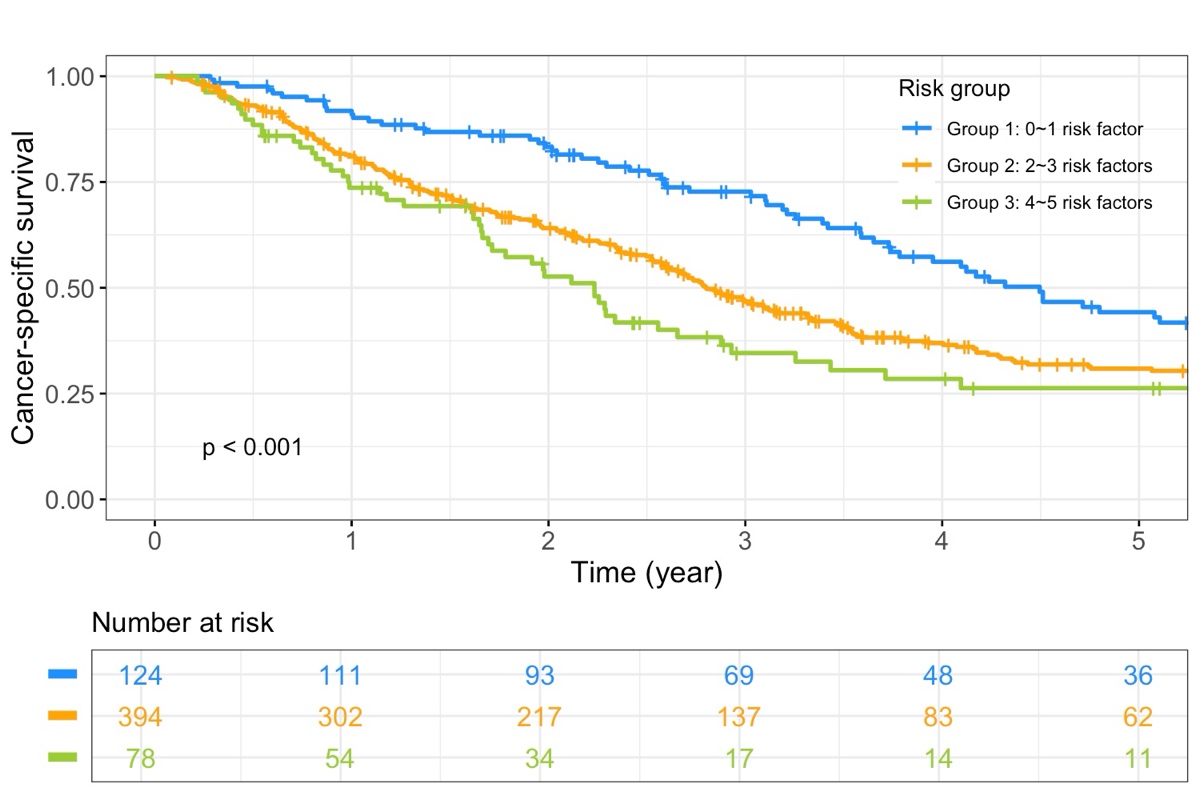
**

**Supplement Table 1 Univariate and multivariate Cox regression analyses of OS and CSS in all UTUC patients**

| Variables | OS | | | | CSS | | | |
| --- | --- | --- | --- | --- | --- | --- | --- | --- |
|  | Univariate | | Multivariate | | Univariate | | Multivariate | |
|  | HR  (95% CI) | p-value | HR  (95% CI) | p-value | HR  (95% CI) | p-value | HR  (95% CI) | p-value |
| Recurrence time | | | | | | | | |
| Late | Reference |  | Reference |  | Reference |  | Reference |  |
| Early | 8.66 (7.33–10.23) | <0.001 | 5.88 (4.90–7.06) | <0.001 | 28.25 (22.88–34.88) | <0.001 | 13.32 (10.50–16.91) | <0.001 |
| Age | | | | | | | | |
| <70 | Reference |  | Reference |  | Reference |  | Reference |  |
| ≥70 | 2.14 (1.90–2.40) | <0.001 | 1.98 (1.76–2.23) | <0.001 | 1.57 (1.29–1.90) | <0.001 | 1.46 (1.19–1.78) | <0.001 |
| Sex | | | | | | | | |
| Male | Reference |  | Reference |  | Reference |  |  |  |
| Female | 0.86 (0.77–0.96) | 0.009 | 0.83 (0.74–0.93) | 0.001 | 0.87 (0.72–1.06) | 0.172 |  |  |
| ECOG | | | | | | | | |
| 0-1 | Reference |  | Reference |  | Reference |  | Reference |  |
| 2-4 | 2.50 (2.04–3.07) | <0.001 | 1.82 (1.47–2.24) | <0.001 | 1.85 (1.27–2.69) | 0.001 | 1.46 (0.99–2.15) | 0.058 |
| DM | | | | | | | | |
| No | Reference |  | Reference |  | Reference |  | Reference |  |
| Yes | 1.43 (1.26–1.62) | <0.001 | 1.16 (1.02–1.32) | 0.025 | 1.28 (1.03–1.60) | 0.026 | 0.95 (0.76–1.19) | 0.649 |
| ESRD/Renal insufficiency | | | | | | | | |
| No | Reference |  | Reference |  | Reference |  |  |  |
| Yes | 1.59 (1.41–1.80) | <0.001 | 1.51 (1.34–1.71) | <0.001 | 1.02 (0.84–1.24) | 0.847 |  |  |
| Smoking | | | | | | | | |
| No | Reference |  |  |  | Reference |  |  |  |
| Yes | 1.12 (0.96–1.31) | 0.134 |  |  | 1.22 (0.95–1.56) | 0.122 |  |  |
| Tumor side | | | | | | | | |
| Unilateral | Reference |  |  |  | Reference |  |  |  |
| Bilateral | 1.35 (0.72–2.52) | 0.345 |  |  | 0.37 (0.05–2.64) | 0.322 |  |  |
| Preoperative hydronephrosis | | | | | | | | |
| No | Reference |  | Reference |  | Reference |  |  |  |
| Yes | 1.26 (1.13–1.41) | <0.001 | 1.03 (0.92–1.16) | 0.578 | 1.63 (1.34–1.99) | <0.001 | 1.25 (1.02–1.53) | 0.031 |
| Tumor location | | | | | | | | |
| Ureter | Reference |  |  |  | Reference |  |  |  |
| Renal pelvis | 0.91 (0.82–1.02) | 0.121 |  |  | 0.94 (0.77–1.14) | 0.53 |  |  |
| Tumor grade | | | | | | | | |
| Low grade | Reference |  | Reference |  | Reference |  | Reference |  |
| High grade/G2 | 1.53 (1.28–1.82) | <0.001 | 1.20 (1–1.44) | 0.046 | 3.98 (2.51­–6.30) | <0.001 | 1.76 (1.09–2.84) | 0.02 |
| Multifocality | | | | | | | | |
| No | Reference |  | Reference |  | Reference |  | Reference |  |
| Yes | 1.53 (1.37–1.71) | <0.001 | 1.18 (1.06–1.33) | 0.004 | 3.46 (2.79–4.28) | <0.001 | 2.08 (1.67–2.60) | <0.001 |
| Lympho-vascular invasion | | | | | | | | |
| No | Reference |  | Reference |  | Reference |  | Reference |  |
| Yes | 1.98 (1.72–2.28) | <0.001 | 1.15 (0.98–1.34) | 0.081 | 3.39 (2.76–4.16) | <0.001 | 1.02 (0.81–1.29) | 0.878 |
| Tumor necrosis | | | | | | | | |
| No | Reference |  | Reference |  | Reference |  | Reference |  |
| Yes | 1.51 (1.29­–1.76) | <0.001 | 1.14 (0.97–1.33) | 0.109 | 2.01 (1.59–2.55) | <0.001 | 1.14 (0.89–1.45) | 0.305 |
| Pathologic T stage | | | | | | | | |
| pTis/pTa/pT1 | Reference |  | Reference |  | Reference |  | Reference |  |
| pT2 | 1.39 (1.20–1.61) | <0.001 | 1.19 (1.02–1.39) | 0.023 | 2.55 (1.85–3.51) | <0.001 | 1.71 (1.23–2.39) | 0.001 |
| pT3 | 2.12 (1.87–2.40) | <0.001 | 1.57 (1.36–1.81) | <0.001 | 7.07 (5.47–9.14) | <0.001 | 3.77 (2.81–5.05) | <0.001 |
| Lymph node dissection | | | | | | | | |
| No | Reference |  |  |  | Reference |  |  |  |
| Yes | 0.92 (0.78–1.07) | 0.277 |  |  | 1.08 (0.84–1.38) | 0.547 |  |  |
| Adjuvant chemotherapy | | | | | | | | |
| No | Reference |  |  |  | Reference |  |  |  |
| Yes | 0.86 (0.72–1.03) | 0.11 |  |  | 1.64 (1.28–2.10) | <0.001 | 0.93 (0.72–1.22) | 0.604 |

**Supplement Table 2 Clinical and pathologic characteristics of development and validation cohort**

| Variables | Development cohort (N=3435) | | | | Validation cohort (N=2246) | | | | | p-value |
| --- | --- | --- | --- | --- | --- | --- | --- | --- | --- | --- |
|  | N | | % | | N | | | % | |  |
| Early recurrence | | | | | | | | | | |
| No | | 3204 | | 93.3 | | 2048 | 91.2 | | 0.003 | |
| Yes | | 231 | | 6.7 | | 198 | 8.8 | |  |  |
| Age | | | | | | | | | | |
| <70 | 1793 | | 52.2 | | 1139 | | | 50.7 | | 0.273 |
| ≥70 | 1642 | | 47.8 | | 1107 | | | 49.3 | |  |
| Sex | | | | | | | | | | |
| Male | 1435 | | 41.8 | | 1515 | | | 67.5 | | <0.001 |
| Female | 2000 | | 58.2 | | 731 | | | 32.5 | |  |
| ECOG | | | | | | | | | | |
| 0 | 2187 | | 63.7 | | 1764 | | | 78.5 | | <0.001 |
| 1 | 1067 | | 31.1 | | 367 | | | 16.3 | |  |
| 2 | 152 | | 4.4 | | 90 | | | 4.0 | |  |
| 3 | 22 | | 0.6 | | 8 | | | 0.4 | |  |
| 4 | 7 | | 0.2 | | 17 | | | 0.8 | |  |
| DM | | | | | | | | | | |
| No | 2655 | | 77.3 | | 1920 | | | 85.5 | | <0.001 |
| Yes | 780 | | 22.7 | | 326 | | | 14.5 | |  |
| Smoking | | | | | | | | | | |
| No | 2899 | | 84.4 | | 1928 | | | 85.8 | | 0.136 |
| Yes | 536 | | 15.6 | | 318 | | | 14.2 | |  |
| Tumor location | | | | | | | | | | |
| Ureter only | 1323 | | 38.5 | | 831 | | | 37.0 | | 0.249 |
| Renal pelvis involvement | 2112 | | 61.5 | | 1415 | | | 63.0 | |  |
| Grade | | | | | | | | | | |
| Low grade | 482 | | 14.0 | | 371 | | | 16.5 | | 0.01 |
| High grade/G2 | 2953 | | 86.0 | | 1875 | | | 83.5 | |  |
| Multifocality | | | | | | | | | | |
| No | 1889 | | 55.0 | | 1435 | | | 63.9 | | <0.001 |
| Yes | 1546 | | 45.0 | | 811 | | | 36.1 | |  |
| Lympho-vascular invasion | | | | | | | | | | |
| No | 2900 | | 84.4 | | 1773 | | | 78.9 | | <0.001 |
| Yes | 535 | | 15.6 | | 473 | | | 21.1 | |  |
| Tumor necrosis | | | | | | | | | | |
| No | 2986 | | 87.0 | | 1765 | | | 78.6 | | <0.001 |
| Yes | 449 | | 13.0 | | 481 | | | 21.4 | |  |
| Pathologic T stage | | | | | | | | | | |
| pTis/pTa/pT1 | 1619 | | 47.1 | | 1086 | | | 48.4 | | 0.593 |
| pT2 | 690 | | 20.1 | | 451 | | | 20.1 | |  |
| pT3 | 1126 | | 32.8 | | 709 | | | 31.6 | |  |
| Lymph node dissection | | | | | | | | | | |
| No | 2747 | | 80.0 | | 1543 | | | 68.7 | | <0.001 |
| Yes | 688 | | 20.0 | | 703 | | | 31.3 | |  |
| Adjuvant chemotherapy | | | | | | | | | | |
| No | 3002 | | 87.4 | | 2043 | | | 91.0 | | <0.001 |
| Yes | 433 | | 12.6 | | 203 | | | 9.0 | |  |
